# Hospital Meetings

**Published:** 1905-06-03

**Authors:** 


### HOSPITAL MEETINGS.

### MIDDLESEX HOSPITAL.

The Governors of the Middlesex Hospital held their quarterly meeting for the transaction of the business of the hospital on the 25th ultimo. Major-General Lord Cheylesmore was reappointed chairman, and Sir Arthur T. Watson, K.C., deputy-chairman of the weekly board for 1905-1906. The Governors agreed to sanction the expenditure of a sum of between £4,000 and £5,000 for necessary repairs to the hospital. Unfortunately while these are taking place it will be necessary for work at the hospital to be suspended for several weeks. A brief report of the transactions of the weekly board having been read over, and the ordinary routine of business disposed of, the chairman made special reference to a resolution which the board had received from the executive committee of King Edward's Hospital Fund to the effect that "The Executive Committee, having considered the recommendation of Sir Edward Fry's Committee, advises the General Council to notify to the hospitals that no grants will be made from the King's Fund, as from and after January 1st, 1906, to any hospitals which make payments to, or on account of their medical schools out of general funds subscribed for the relief of the sick poor." As since its foundation in 1835 the medical school has formed an integral part of the hospital, and has been partially supported by the latter's subscription funds, the Governors have decided to convene a special meeting for the discussion of the very important question as to how the school may continue to be maintained without the hospital sustaining the loss of a grant from the King's Fund. This meeting will take place in about a month's time.

Amongst those attending on the present occasion were Major-General Lord Cheylesmore (in the chair), Major-General Sir Reginald T. Thynne, K.C.B., Sir Arthur Townley Watson, K.C., Lieutenant-Colonel J. Lambert Mears, M.A., LL.D., Horace Cockerell, Esq., Dr. William Cayley, C. H. Burnand, Esq. (treasurer).

The proceedings were concluded with a vote of thanks to the chairman.

### THE BROMPTON HOSPITAL FOR CONSUMPTION.

A meeting of the Court of Governors of the Brompton Hospital for Consumption took place in the board room of the hospital on May 26th. Major-General Lord Cheylesmore took the chair, and the usual précis of the hospital's recent transactions was laid before the Governors present.

The report was adopted. The figures submitted unfortunately show a decrease both in legacies and donations, and the Governors wish to make an earnest appeal for funds for the support of the hospital's new sanatorium near Frimley, which, as readers will recollect, was formally opened by the Prince and Princess of Wales on June 25th last. It is now in working order and has at present 50 patients who have been drafted to it from the hospital, but as the annual cost of maintaining the sanatorium is about £10,000, the urgent need for public financial assistance is apparent. Mention was also made of the Governors' regret that the Council of King Edward's Hospital Fund, though expressing their satisfaction at the work of the hospital, gave no grant except £350 for ward furniture.

The Court of Governors appointed successors to the posts of the several members of the medical staff who had resigned, mostly after long and faithful service, to which the chairman made sympathetic reference. Foremost mention must be made of the 50 years' devoted and successful work in the hospital's interests which is the record of Mr. William H. Theobald, the now retiring secretary-superintendent. Dr. Fowler, the senior physician, has also resigned his position after 25 years' excellent service.

Reference was made to the success of the new Jewish ward, opened some time ago by Dr. Adler, where Jewish patients have their food prepared according to the rites of their own religion.

The Court of Governors also elected a Committee of Management, and a lady almoner has for the first time been appointed.

A vote of thanks having been passed to Lord Cheylesmore for presiding, the meeting terminated.

### HOSPITAL FOR SICK CHILDREN, GREAT ORMOND STREET.

The Duke of Fife presided at the fifty-third annual meeting of the Hospital for Sick Children on May 25th. His Grace pointed out that, like most hospitals, they needed more annual subscribers, and, if possible, a larger endowment fund. He foresaw a time when, if the public did not support charitable institutions of this kind more liberally, Government would be compelled to take them over. It was a sad fact that the generality of the public contributed little or nothing to hospitals, and it was left to a few only, who gave generously and lavishly. He considered that this state of things was brought about by the condition of apathy in which the world was sunk as to the needs and requirements of hospitals. The work done at the Children's Hospital was far-extending. In the past year 2,000 in-patients and 30,000 out had been treated. It was most satisfactory to think of the alleviation of suffering represented by these figures, but they were always confronted by the gaunt spectre of debt. His Grace concluded by moving the adoption of the report, which was unanimously accepted.

The chairman, Mr. Arthur Lucas, spoke at some length on the subject of finance. Since H.R.H. the Prince of Wales had uttered some words of warning on the question of expense, the Managing Committee had done their best to see if there was any way in which they could lessen expenditure, with the result that they had succeeded in lessening the outlay on each patient by 5s. 1d., and, with some other economies, had reduced expenditure in 12 months by £1,335. It was unavoidable that as science revealed new ways of lessening disease and suffering the cost of maintaining a hospital in working order, with all possible up-to-date arrangements, became greater; hence the need for more money. Another point on which Mr. Lucas spoke with great force was that of the rating of the hospital. This amounted to £800 a year. Nearly one shilling in every pound thus goes to parish rates.

Proceedings terminated with a vote of thanks to the medical officers, to the honorary auditors, and to his Grace the Duke of Fife for presiding at the meeting.

### THE HOSPITAL FOR EPILEPSY AND PARALYSIS.

The annual meeting of the Hospital for Epilepsy and Paralysis was held at the new hospital in Maida Vale on May 26th, the vice-president, Viscount Clifden, being in the chair.

The Chairman moved that the report and statement of accounts should be adopted; Canon Barker seconded the resolution, and it was passed unanimously. The Chairman drew attention to the fact that, so far, in spite of the heavy expenses of building and moving into the new premises, the hospital had steered clear of debt. Some people, he said, were of opinion that it was well that hospitals should always be more or less in debt, because it moved the public heart; but he could not think that this was either wise or right. There was much cause for thankfulness, inasmuch as £2,261 had been gained from legacies and £250 from the proceeds of entertainments; this timely help prevented a further necessary sale of stock, a thing always to be deplored. King Edward's Hospital Fund had contributed £400, the Hospital Saturday Fund £40, and the Hospital Sunday Fund £53 13s. 4d. The great need is for annual subscriptions, which should be the "backbone" of every charitable institution. These only amount to £311, one-eighth only of the total expenditure for the year.

Viscount Clifden alluded to the loss they had sustained in the lamented death of the president, Lord Hardwicke, and also of the vice-president, Lord Northbrook.

Canon Barker spoke on several points, especially criticising the position of charities with regard to the heavy rates that they are compelled to pay. It was, he thought, unjustifiable that such an institution as that on whose behalf they had met together should have to pay £219 10s. 8d. for rates and taxes. He noted with satisfaction that patients able to pay were expected to contribute something, according to their means, to the expenses of their treatment and keep, and he thought that such an arrangement might well be more generally adopted in hospitals.

A vote of thanks having been given to Viscount Clifden for presiding the meeting terminated.

